# Identification of symptom and functional domains that fibromyalgia patients would like to see improved: a cluster analysis

**DOI:** 10.1186/1471-2474-11-134

**Published:** 2010-06-28

**Authors:** Robert M Bennett, Jon Russell, Joseph C Cappelleri, Andrew G Bushmakin, Gergana Zlateva, Alesia Sadosky

**Affiliations:** 1 Oregon Health & Science University, Portland, Oregon, USA; 2 University of Texas Health Science Center at San Antonio, San Antonio, Texas, USA; 3 Pfizer Global Research and Development, New London, Connecticut, USA; 4 Pfizer Global Outcomes Research, New York, New York, USA

## Abstract

**Background:**

The purpose of this study was to determine whether some of the clinical features of fibromyalgia (FM) that patients would like to see improved aggregate into definable clusters.

**Methods:**

Seven hundred and eighty-eight patients with clinically confirmed FM and baseline pain ≥40 mm on a 100 mm visual analogue scale ranked 5 FM clinical features that the subjects would most like to see improved after treatment (one for each priority quintile) from a list of 20 developed during focus groups. For each subject, clinical features were transformed into vectors with rankings assigned values 1-5 (lowest to highest ranking). Logistic analysis was used to create a distance matrix and hierarchical cluster analysis was applied to identify cluster structure. The frequency of cluster selection was determined, and cluster importance was ranked using cluster scores derived from rankings of the clinical features. Multidimensional scaling was used to visualize and conceptualize cluster relationships.

**Results:**

Six clinical features clusters were identified and named based on their key characteristics. In order of selection frequency, the clusters were Pain (90%; 4 clinical features), Fatigue (89%; 4 clinical features), Domestic (42%; 4 clinical features), Impairment (29%; 3 functions), Affective (21%; 3 clinical features), and Social (9%; 2 functional). The "Pain Cluster" was ranked of greatest importance by 54% of subjects, followed by Fatigue, which was given the highest ranking by 28% of subjects. Multidimensional scaling mapped these clusters to two dimensions: Status (bounded by Physical and Emotional domains), and Setting (bounded by Individual and Group interactions).

**Conclusion:**

Common clinical features of FM could be grouped into 6 clusters (Pain, Fatigue, Domestic, Impairment, Affective, and Social) based on patient perception of relevance to treatment. Furthermore, these 6 clusters could be charted in the 2 dimensions of Status and Setting, thus providing a unique perspective for interpretation of FM symptomatology.

## Background

Chronic widespread pain is the primary characteristic of FM. Its presence is the essential feature of the current American College of Rheumatology guidelines for the classification of FM [1] and it is the best understood feature of this syndrome [2,3]. In addition to pain, FM patients typically exhibit a number of co-morbidities that limit productivity, personal and family life, and activities of daily living [4-8]. These comorbidities may include fatigue, sleep disturbance, mood disorders, cognitive impairment, restless legs, balance problems, recurrent headaches, irritable bowel, and overactive bladder [9]. This heterogeneity has contributed to difficulty in characterizing the inter-relationships among FM clinical features and in determining the importance of various comorbidities to patients and their treatment outcomes.

Cluster analysis is a multivariate statistical technique that can be used for evaluating the degree of similarity among apparently heterogeneous variables, and identifying related groups of variables based on these similarities. The concept of identifying clusters of patient-relevant clinical features through cluster analysis has become important in the oncology setting as a tool for developing patient orientated management strategies [10,11]. This concept has been applied to other disorders in an effort to understand the relationships among clinical features and outcome variables [12-14].

Cluster analysis has also been applied to FM in several studies [15-20]. While some of these studies categorized patients through the use of objective quantitative sensory testing [15] and psychophysiologic response patterns [18], others identified subgroups based on the presence and severity of physical and/or affective clinical features [16,19,20]. In contrast to these studies, which have used cluster analysis to identify *patient *clusters (i.e., groups of patients with similar characteristics), we have attempted to identify clusters of *clinical features *that are meaningful to FM patients and correspond to their treatment priority goals in the context of desired improvement.

In several ways, a cluster analysis based on clinical features extends beyond a cluster analysis of patients who share a similar clinical profile. This type of analysis directly targets, aggregates, and identifies those clinical features that are related (i.e. cluster together); quantifies the importance of each cluster (i.e., which clusters are more clinically relevant); and allows interpretation of clusters with respect to their perceptual relationships and key attributes (i.e., what characterizes the different clusters).

As part of an approach to understanding the relationships among the clinical features of FM, we focused on identifying those features that patients would most like to be improved as a result of treatment, and then aggregated those preferences into clusters. The study reported here describes a cluster analysis algorithm for identifying patient-relevant groups of closely-related clinical features. The intent of this exercise is to: 1) promote a better understanding of FM through grouping clinical features (rather than individual features in isolation), and 2) characterize key clusters that may facilitate the assessment and management of FM.

## Methods

This study is a secondary analysis using baseline data of patients with FM, before randomization to either pregabalin or placebo, who were entered into a double-blind placebo-controlled randomized clinical trial of pregabalin. Details of the trial and the treatment groups have previously been published [21]. As this was a secondary analysis, research subjects' anonymity was preserved and an ethical committee approval was not required; the study was conducted in accordance with the Declaration of Helsinki. All subjects met the 1990 ACR definition for FM [1] and had a pain rating ≥40 mm on a 100 mm visual analogue scale (VAS). They were asked to identify 5 FM clinical features, from a list of 20 distinct clinical features, which they would most like to see improved after treatment. The list of clinical features used in this study was developed during patient focus groups that were conducted to elicit concepts relevant to assessing the impact of FM on their lives [22].

After identifying the 5 clinical features, subjects then assigned each clinical feature a relative importance by arranging them in order within five priority quintiles. The rationale for choosing, a priori, the top 5 clinical features comes from the demonstration in cognitive psychology that the upper limit of categories that individuals can discriminate is near 7 plus or minus 2 [23,24].

### Distance Matrix

The analysis was initiated by representing each subject by a vector of 20 values (one for each clinical feature), with the rankings assigned values of 1-5 (lowest to highest ranking; clinical features not chosen were assigned a value of 0) for the 5 most important FM clinical features.

We began to formally evaluate the relationships between the clinical features by using logistic regression [25] to predict the likelihood of selecting each clinical feature based on the selection of another clinical feature, and thus deriving an odds ratio for the likelihood of each pair of features being co-selected. This method essentially created a distance matrix between the pairs based on rankings. For example, the clinical feature "pain or discomfort" (item 15) and the clinical feature "disturbed sleep" (item 16) can then be used to create a 2-by-2 cross-classification table to quantify the relationship between them: 1 means that the clinical feature was selected among a subject's five most important clinical features, and 0 means that it was not ranked in the top five.

The odds ratio of having selected a second variable after a particular first variable was calculated for all possible pair-wise combinations of clinical features to define the relative distance between co-selected variables. The inverse or reciprocal of each of the resulting odds ratios was then calculated (so that a smaller odds ratio would be represented by a greater distance) to serve as proxies to define distances between clinical features as input into the distance matrix between the pairs in the cluster analysis.

### Cluster Analysis

Based on the distance matrix between pairs of clinical features, a hierarchical cluster analysis [26] was the algorithm applied to identify cluster structure. This hierarchical cluster analysis was used to group together clinical features that are "close to one another" into a cluster such that those clinical features within each cluster are more closely related to one another than clinical features assigned to different clusters. In hierarchical clustering, a major method of clustering, the data are not partitioned into a particular cluster in a single step. Instead, a series of partitions takes place, which may run from a single cluster containing all clinical features to many clusters, each containing a single clinical feature.

Based on the cluster algorithm, a dendogram was generated for visual classification of similarity for grouping the clinical features. In the dendogram, the clinical features were represented as nodes and the branches illustrated when the cluster method joined subgroups of clinical features. The length of the branch indicated the distance between the subgroups of clinical features when they were joined. The cluster analysis also identified clusters of those clinical features concurrently selected by the subjects. The frequency of cluster selection was determined and cluster importance was ranked. Cluster importance score was defined by the selection frequencies of the individual clinical features adjusted for the number of clinical features in a cluster.

### Multidimensional Scaling

Multidimensional scaling is a multivariate statistical technique often used to visualize information for exploring similarities or dissimilarities in data [27], and it was utilized here to discern and explore the similarities and dissimilarities among the clinical features. Based on the distance between clinical features in the cluster analysis, multidimensional scaling was applied to characterize the perceptual relationship among clusters, and to provide insight and interpretation to the clusters by presenting and relating them in terms of meaningful and understandable descriptors. The names that were eventually given to each of the clusters were determined post-hoc in an attempt to characterize the nature of each cluster in perceptual space. In doing so, the geometric results from the multidimensional scaling, which provided a perceptual map but no clusters, were used to complement and enhance the results of the cluster analysis, which provided clusters but no perceptual map.

All analyses were performed in SAS^®^, including its procedures PROC LOGISTIC, PROC CLUSTER and PROC MDS [28].

## Results

Rankings from a total of 788 subjects were surveyed. The subjects were predominantly female (95%), with a mean age of 50 years and a mean duration of FM of 10 years. Of the 788 subjects, 34 (4.3%) chose not to rank clinical features and were assigned rankings of zero for all 20 items.

The 20 clinical features and the proportion of subjects ranking their importance are shown in Table 1. Pain was ranked as the most important clinical feature by the greatest proportion of subjects (43.9%). It was followed in the first priority quintile by lack of energy (17%), disturbed sleep (10%), feeling tired (6%), and difficulty thinking (5%). The clinical features ranked as second in importance (second quintile) were disturbed sleep (21%), followed by lack of energy (17%), pain or discomfort (15%), feeling tired (15%) and difficulty thinking (6%).

**Table 1:** Ranking of treatment-relevant clinical features among 788 fibromyalgia patients*

Symptom Number	Symptom	Rank order by importance, n (%) Priority Quintiles					
		
		**1**^**st**^	**2**^**nd**^	**3**^**rd**^	**4**^**th**^	**5**^**th**^	Not ranked
q1	Having a lack of energy	126 (16.0)	129 (16.4)	106 (13.5)	70 (8.9)	68 (8.6)	289 (36.7)
q2	Having to push yourself to do things	18 (2.3)	39 (4.9)	69 (8.8)	67 (8.5)	65 (8.3)	530 (67.3)
q3	Interference with your ability to accomplish daily tasks, such as housework or shopping	16 (2.0)	16 (2.0)	42 (5.3)	41 (5.2)	52 (6.6)	621 (78.8)
q4	Impact on your family	33 (4.2)	17 (2.2)	20 (2.5)	26 (3.3)	29 (3.9)	663 (84.1)
q5	Difficulty being sexually intimate with your partner	9 (1.1)	15 (1.9)	24 (3.1)	30 (3.8)	29 (3.7)	681 (86.4)
q6	Feeling tired	43 (5.5)	111 (14.1)	111 (14.1)	95 (12.1)	61 (7.7)	367 (46.6)
q7	Interference with work or school	11 (1.4)	6 (0.8)	17 (2.2)	21 (2.7)	22 (2.8)	711 (90.2)
q8	Feeling anxious	2 (0.3)	9 (1.1)	3 (0.4)	14 (1.8)	17 (2.2)	743 (94.3)
q9	Difficulty thinking or "fibro fog"	37 (4.7)	44 (5.6)	62 (7.9)	89 (11.3)	68 (8.6)	488 (61.9)
q10	Feeling like the pace of your life is slower than most other people	1 (0.1)	8 (1.0)	7 (0.9)	10 (1.3)	15 (1.9)	747 (94.8)
q11	Impact on your social life	1 (0.1)	4 (0.5)	5 (0.6)	10 (1.3)	16 (2.0)	752 (95.4)
q12	Driving limitations	0	1 (0.1)	1 (0.1)	0	5 (0.6)	781 (99.1)
q13	Skin is sensitive to touch	6 (0.8)	12 (1.5)	16 (2.0)	25 (3.1)	34 (4.3)	695 (88.2)
q14	Feeling depressed	11 (1.4)	15 (1.9)	21 (2.7)	31 (3.9)	29 (3.7)	681 (86.4)
q15	Pain or discomfort	329 (41.8)	116 (14.7)	80 (10.2)	62 (7.9)	43 (5.5)	158 (20.1)
q16	Disturbed sleep	71 (9.0)	159 (20.2)	103 (13.1)	77 (9.8)	58 (7.4)	320 (40.6)
q17	Strain on your relationship with your spouse	8 (1.0)	10 (1.3)	16 (2.0)	13 (1.7)	23 (2.9)	718 (91.1)
q18	Feeling isolated or alone	2 (0.3)	1 (0.1)	6 (0.8)	8 (1.0)	21 (2.7)	750 (95.2)
q19	Being unable to make plans with the confidence that you will follow-through	5 (0.6)	16 (2.0)	16 (2.0)	19 (2.4)	55 (7.0)	677 (85.9)
q20	Difficulty walking	19 (2.4)	22 (2.8)	27 (3.4)	43 (5.5)	40 (5.1)	637 (80.8)

* The left column indicates the question number while the second column provides an abstraction of the original question regarding a specific clinical feature. The five columns to the right contain the number and proportion of subjects who ranked that question as 1^st^, 2^nd^, 3^rd^, 4^th ^or 5^th ^in importance, respectively.

Since the two clinical features ranked overall as first and second in importance were respectively "pain or discomfort" (q15) and "disturbed sleep" (q16), it is illustrative to note how their relationship was determined for use in the cluster analysis. Those two clinical features were each selected in the top five by 401 patients (these patients were scored 1 for "pain or discomfort" and 1 for "disturbed sleep"). Neither of these two features was selected in the top five by 91 patients (these patients were scored 0 for "pain or discomfort" and 0 for "disturbed sleep"). "Pain or discomfort" was selected in the top five but disturbed sleep was not selected in the top 5 by 229 patients (these patients scored 1 for "pain or discomfort" and 0 for "disturbed sleep") while the reverse occurred in 67 patients (these patients were scored 0 for "pain or discomfort" and 1 for "disturbed sleep"). Therefore, the odds of having selected "pain or discomfort," if "disturbed sleep" was selected, is 401*91/229*67, or approximately 2.4-times as likely relative to "disturbed sleep" not being selected. The relationships for other pairs of clinical features were similarly assessed for input into the distance matrix in the cluster analysis.

**Figure 1:**
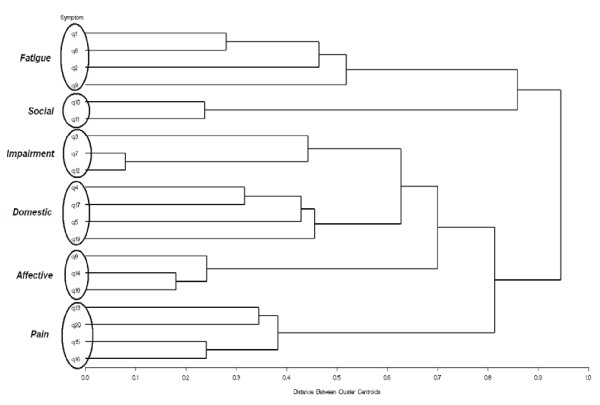
**Dendogram from the cluster analysis of the 20 clinical features**. The dendrogram was generated using odds ratios (i.e. likelihood of pairs of symptoms being co-selected) as a proxy for distance between symptoms. A description of each clinical feature by number is presented in Table 1.

Clustering identified 6 distinct groups, with each cluster having several related clinical features (Figure 1). These six clusters were named on the basis of their key characteristics. They included the following: 1) "Fatigue" (composed of clinical features q1, q2, q6, and q9), 2) "Social" (clinical features q10 and q11), 3) "Impairment" (clinical features q3, q7, and q12), 4) "Domestic" (clinical features q4, q5, q17, and q19), 5) "Affective" (clinical features q8, q14, and q18), and 6) "Pain" (clinical features q13, q15, q16, and q20).

**Figure 2:**
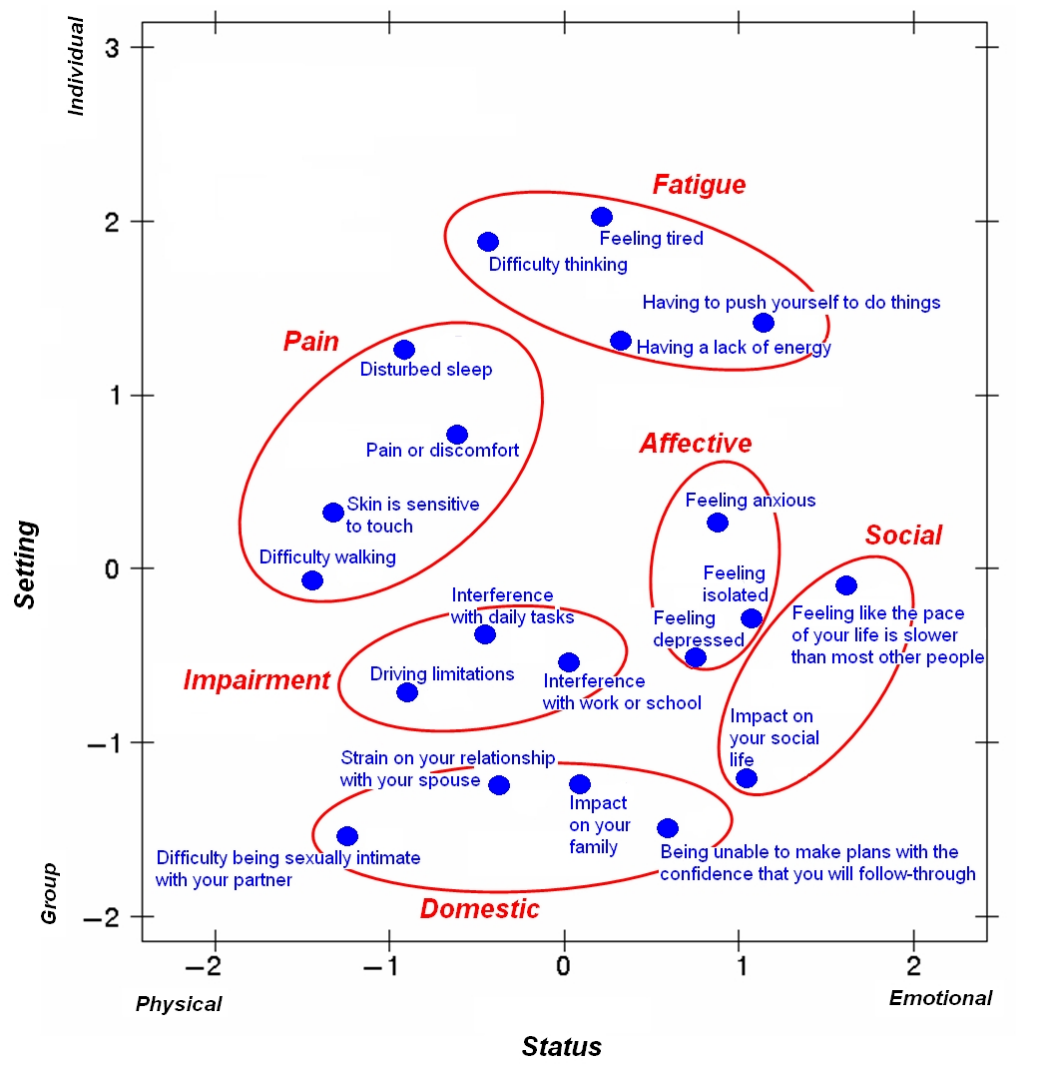
**Multidimensional scaling map of the conceptual relationships among the clusters of clinical features**. The conceptual map shows patient perceptions of the 20 clinical features divided into 6 distinct clusters on the dimensions of Setting, bounded by Group and Individual interactions on the vertical axis, and Status, bounded by Physical and Emotional domains on the horizontal axis. The items in each cluster were determined by the odds ratio of each item being co-selected by fibromyalgia patients as being important to their care. The relative location of each cluster identifies its dimensional relationship. For example, the Pain cluster would be considered strong with regard to Individual effects and the Physical domain, while the Domestic cluster would be strongly Group-related but would have roots in both Physical and Emotional domains.

Multidimensional scaling performed on the distance between clinical features confirmed the conceptual validity of the proposed clusters and provided a graphic representation of how the clusters are perceptually related (Figure 2). As shown in Figure 2, the conceptual map shows patient perceptions of the 20 clinical features grouped into six distinct clusters that spread across two dimensions. These dimensions were defined as "Setting" (vertical axis, bounded by group and individual interactions) and "Status" (horizontal axis, bounded by physical and emotional domains). This two-dimensional configuration was found to be statistically and substantially sufficient in accordance with the goal of emphasizing a parsimonious and substantive interpretation consistent with a rigorous empirical analysis. Calculations using three or more dimensions did not improve the fit of the model.

For example, the analysis suggested that this sample of patients felt that clinical features in the Social cluster (bottom right) impinged on group-oriented activities and had the highest emotional consequence. Symptoms in the Domestic cluster (bottom center) were perceived as adversely affecting group interaction the most and impacting both physical and emotional factors. Symptoms in the Fatigue cluster (top) were rated as being most intrusive to the individual and having both physical and emotional implications. Symptoms in the Impairment cluster (center) were thought to interfere with physical activities, have emotional impact, and affect group interactions. Symptoms in the Affective cluster (center right) occurred in both individual and group settings with substantial emotional consequences. Symptoms in the Pain cluster (top left) compromised the individual with predominantly physical limitations.

A complementary way to interpret Figure 2 is in terms of cluster similarity and dissimilarity. For example, clinical features of Fatigue were in closer proximity--and hence more related--to clinical features of Pain than to clinical features covering Domestic issues.

The clusters most frequently chosen, based on the cluster importance score greater than 0, were those related to the physical domains (Table 2). Symptoms contained within the Pain Cluster were selected by 90.1% of subjects, closely followed by those in the Fatigue Cluster (89.3%). The most commonly endorsed psychosocial domain cluster was Domestic (42.0%), followed by Affective (20.9%); the Social Cluster was selected by only 9.3% of subjects. In addition to being the most frequently invoked cluster, the Pain Cluster was also ranked the most important cluster by the highest proportion of patients (53.9%), and ranked second in importance by another 14.7% of subjects (Table 2). In contrast, the Fatigue Cluster was ranked as first and second most important by an approximately equal proportion of patients (28.4% and 27.9%, respectively). The psychosocial domain clusters (Affective, Domestic, and Social) were more likely to be selected as the least important clusters.

**Table 2:** Selection and ranking frequency of treatment-relevant clusters of clinical features.*

Cluster	Selection frequency, % of subjects	Ranking frequency, % of subjects					
		**1**	**2**	**3**	**4**	**5**	**Not ranked**

Pain	90.1	53.9	14.7	11.6	6.5	3.4	9.9
Fatigue	89.3	28.4	27.9	20.9	8.0	4.1	10.7
Domestic	42.0	7.0	6.0	8.1	7.7	13.2	58.0
Impairment	29.1	3.4	2.7	7.2	7.0	8.8	70.9
Affective	20.9	1.9	2.9	3.3	6.1	6.7	79.1
Social	9.3	0.3	1.5	1.5	2.0	3.9	90.7

*For frequency, cluster was marked as selected if at least one clinical feature from this cluster was chosen. Ranking was determined based on the maximum number of clinical features scored from every cluster.

## Discussion

The multidimensional nature of FM obscures our understanding of the burden of FM in individual patients and complicates the development of appropriate comprehensive management strategies. Understanding the full complexity of the clinical features of FM is becoming increasingly important with the availability of newer pharmacologic therapies that have significant effects on pain and other clinical features such as sleep (pregabalin [21], sodium oxybate [29,30]), affective symptoms (duloxetine [31,32]), and fatigue and cognitive function (milnacipran [33]. Additionally, recent proposals suggesting the use of combination therapies [34-36] warrant a more complete understanding of the relationships among clinical features from the patient perspective.

The approach described here measured the strength of the relationship among the 20 clinical features that FM patients would most like to be improved during treatment. It was found that these clinical features could be categorized into 6 clusters. Furthermore, these clusters could be mapped in the two dimensions of Status (horizontal axis, bounded by physical and emotional domains) and Setting (vertical axis, bounded by group and individual interactions). These 2 dimensions represent how patients perceive the impact of their clinical features with respect to emotional and physical consequences (Status), as well as whether they affect individual or group interactions (Setting). Not surprisingly, these clusters are similar to the four domains of physical, cognitive/emotional, social, and work/activity, identified during the focus groups which served as the foundation for developing the 20 clinical features for ranking [22]. However, in contrast to the focus groups, our cluster analysis identified Pain and Fatigue as independent clusters. The Pain Cluster was predominantly associated with the physical domain, while the Fatigue Cluster had both physical and emotional components. The Impairment Cluster, which relates to an FM-related inability to perform daily tasks, was also identified as being independent from the other 5 clusters.

Interestingly, "Disturbed sleep (q9)" clustered with pain rather than fatigue. While pain and sleep have been suggested to have a reciprocal relationship in chronic pain conditions including FM [37-39], our results raise the question as to whether pain causes disturbed sleep or pain is a result of disturbed sleep, as originally proposed by Moldofsky et al. [40] and further supported by recent studies [41,42]. Of similar interest, "Difficulty thinking or fibro-fog (q9)" clustered with the physical domain cluster of Fatigue. Although the significance of this clustering is presently unclear, it is noteworthy that Katz et al. [43] reported an association of "fibrofog" with increased symptom intensity in terms of pain, stiffness, fatigue, and disturbed sleep.

Our use of cluster analysis to identify *clinical features *clusters is in contrast to other studies which used the same technique to identify *patient *clusters [15-20]. While these two approaches can provide complementary information that can aid in our understanding of FM, clinical features clusters may provide a more clinically relevant approach since current management goals are clinical features-driven.

The concept of ranking FM treatment goals has previously been explored using cluster analysis in a study by Hamilton et al. [17]. They identified 3 subgroups based on the patients' ranking of FM-specific goals. These subgroups included seeking professional care, which may correlate to the physical domain clusters and the Affective cluster in the current study; self-sufficiency goals, which may correlate with our Impairment cluster; and social-validation goals, which likely correlates with the those clusters affecting group interactions. Their demonstration that goal profiles covaried with differences in pain, negative affect, goal-specific social support, general social support, goal-related interference, and negative life events provide further support for the use of clinical features clusters as another means of determining patient priorities.

Although the frequency with which the Pain cluster and Fatigue cluster were identified by subjects as being important was comparable, the Pain cluster was selected as most important by 54% of subjects, consistent with pain being the hallmark clinical feature of FM [1]. The Fatigue cluster was selected as most important by 28% of subjects, but the psychosocial clusters were not considered to be as high a priority, with ≥70% of subjects not giving them a ranking.

A recent study that used exploratory factor analysis to evaluate correlations among 20 symptoms reported by patients with FM suggested that 17 of these symptoms partition into 5 factors or clusters: Somatic (extremity swelling, rashes, restless legs, abdominal pain, bladder problems, muscle spasms), Distress (anxiety, depression), FM Core (morning stiffness, pain, fatigue), Dyscognition (postural instability, dizziness, concentration problems, forgetfulness), and Sleep Problems (inability to fall asleep, inability to stay asleep) [44]. However, that study was substantially different from ours not only in their use of factor analysis rather than cluster analysis, but also with regard to their perspective in choice of symptoms. In contrast to the current study, which utilized a patient perspective, they took a more clinical perspective by choosing symptoms that were reported as being present among a surveyed population of patients with FM. Additionally, they used symptom severity as the basis for their correlation analysis, which probably accounts for their reported grouping of symptoms within cluster; the presence and severity of a particular symptom may not necessarily equate with patients' preferences for its treatment.

Because of limitations inherent in our investigation, care and context should be exercised in interpreting the results of this study. Some of these limitations are represented by the inclusion/exclusion criteria, which can be construed as limiting the application of our results to the more general clinical FM population. In particular, there was an inclusion requirement for a minimum pain severity score, and a criterion of exclusion for subjects with major depressive disorder. However, not only is the pain requirement consistent with clinical studies evaluating new FM therapies, but pain is the major clinical feature of FM, and despite inter-patient variability in pain severity scores [45], a mean pain score of 6.4 ± 2.0 has been reported from FM subjects in the general population [9], suggesting that the minimum severity level required for this study is not an unreasonable expectation in clinical practice. Although subjects with major depressive disorder were excluded, depression is present in about 34% of FM patients [46]. In the current population, baseline scores of score 8.7 ± 4.2 for anxiety and 7.1 ± 4.1 for depression on the Hospital Anxiety and Depression Scale, indicated the presence of at least mild depression and anxiety [21].

The input for the cluster analysis was derived from the relative importance of the five most bothersome clinical features that a subject would like to see addressed after entry into an industry-supported pharmacologic study. This aspect of the study may be criticized as a limitation, since patients' expectations with regard to their treatment in a clinical trial could have potentially introduced bias in their responses. Nevertheless, given that the chosen clinical features stem from those documented as having a substantial negative impact on daily life, based on focus groups [22], the same selection of clinical features would likely be made if patients were simply asked about the most problematic clinical features they experience. Our findings document and quantify that pain is the most prominent clinical feature for FM patients, with fatigue being the next most problematic symptom; this confirms the findings of other studies [20,47,48].

Of note, stiffness, which has recently been recognized as an important symptom for FM patients [47], albeit not as part of the core or secondary domains required for assessment in clinical trials [49], does not appear in any of our identified clusters. The reason for this omission is that stiffness, despite being of clinical importance, was not among those clinical features identified as being of high relevance to patients in the focus groups [22], and was thus omitted from the list of 20 clinical features presented to the subjects in the current study.

Ongoing study of multiple clinical features within and between clusters will yield empirical confirmation or refutation on the existence and extent of these clusters. For instance, a recent factor analysis of musculoskeletal, non-musculoskeletal, and cognitive/psychological domains derived from the National Fibromyalgia Association patient survey revealed 4 distinctive clusters [50]. Cluster 1 was high on all 3 domains, cluster 2 was moderate on musculoskeletal/non-musculoskeletal domains and high on cognitive/psychological domains, cluster 3 was moderate on the musculoskeletal/non-musculoskeletal domains and low on the cognitive/psychological domain, and cluster 4 was low on all 3 domains. It was concluded that the reporting of clinical features in FM patients is quite variable, and that increased understanding of subgroups in relation to disease mechanisms may provide useful guidance for treatment decisions.

## Conclusion

This study characterizes the distinctive clustering of clinical features that FM patients would like to see improved. These clinical features grouped into 6 clusters with two separate clusters, Pain and Fatigue, being the most frequently invoked clusters. While both clusters impacted the individual, Pain was related to the physical domain and Fatigue spread across physical and emotional domains. Pain and Fatigue were given the highest ranking in importance by the greatest proportion of patients: 54% for the Pain Cluster and 28% for the Fatigue Cluster. Further evaluation is warranted to determine the clinical relevance of clinical features clusters to the diagnosis and treatment of fibromyalgia.

## Abbreviations

FM: Fibromyalgia; VAS: Visual analog scale; SAS^®^: Statistical Analysis Software.

## Competing interests

RMB and IJR are consultants to Pfizer, Inc. and have received research support and honoraria from Pfizer, Inc. JCC, AGB, GZ and AS are all employees and hold stock in Pfizer, Inc.

## Authors' contributions

RMB and IJR assisted with the conceptualization and editing of the manuscript, clinical guidance, research of the disease area, interpretation of results, and conclusions. JCC and AGB conceived the study design, performed the data analysis, interpreted the results, contributed to the conclusions, and edited the manuscript. AS and GZ assisted with development of manuscript outline, data interpretation, subject matter content, and conclusions. All authors were involved in discussion of the findings, as well as in the drafting and final approval of the manuscript.

## Pre-publication history

The pre-publication history for this paper can be accessed here:

http://www.biomedcentral.com/1471-2474/11/134/prepub

## References

[B1] Wolfe F Smythe HA Yunus MB Bennett RM Bombardier C Goldenberg DL Tugwell P Campbell SM Abeles M Clark P The American College of Rheumatology 1990 Criteria for the Classification of Fibromyalgia. Report of the Multicenter Criteria Committee Arthritis Rheum 1990 33 2 160 172 10.1002/art.1780330203 2306288

[B2] Bennett RM Emerging concepts in the neurobiology of chronic pain: evidence of abnormal sensory processing in fibromyalgia Mayo Clinic Proc 1999 74 385 398 10.4065/74.4.385 10221469

[B3] Staud R Evidence of involvement of central neural mechanisms in generating fibromyalgia pain Curr Rheumatol Rep 2002 4 299 305 10.1007/s11926-002-0038-5 12126581

[B4] White KP Speechley M Harth M Ostbye T Comparing self-reported function and work disability in 100 community cases of fibromyalgia syndrome versus controls in London, Ontario. The London Fibromyalgia Epidemiology Study Arthritis Rheum 1999 42 1 76 83 10.1002/1529-0131(199901)42:1<76::AID-ANR10>3.0.CO;2-G 9920017

[B5] Verbunt JA Pernot DH Smeets RJ Disability and quality of life in patients with fibromyalgia Health Qual Life Outcomes 2008 6 8 10.1186/1477-7525-6-8 18211701 PMC2265693

[B6] Berger A Dukes E Martin S Edelsberg J Oster G Characteristics and healthcare costs of patients with fibromyalgia syndrome Int J Clin Pract 2007 61 9 1498 1508 10.1111/j.1742-1241.2007.01480.x 17655684 PMC2040193

[B7] Hoffman DL Dukes E The health status burden of people with fibromyalgia: a review of studies that assessed health status with the SF-36 or the SF-12 Int J Clin Pract 2008 62 1 115 126 10.1111/j.1742-1241.2007.01638.x 18039330 PMC2228371

[B8] White LA Birnbaum HG Kaltenboeck A Tang J Mallett D Robinson RL Employees with fibromyalgia: medical comorbidity, healthcare costs, and work loss J Occup Environ Med 2008 50 1 13 24 10.1097/JOM.0b013e31815cff4b 18188077

[B9] Bennett RM Jones J Turk DC Matallana L An internet survey of 2,596 people with fibromyalgia BMC Musculoskelet Disord 2007 8 27 10.1186/1471-2474-8-27 17349056 PMC1829161

[B10] Barsevick AM The elusive concept of the symptom cluster Oncol Nurs Forum 2007 34 5 971 980 10.1188/07.ONF.971-980 17878126

[B11] Barsevick AM Whitmer K Nail LM Beck SL Dudley WN Symptom cluster research: conceptual, design, measurement, and analysis issues J Pain Symptom Manage 2006 31 1 85 95 10.1016/j.jpainsymman.2005.05.015 16442485

[B12] Ryan CJ DeVon HA Horne R King KB Milner K Moser DK Quinn JR Rosenfeld A Hwang SY Zerwic JJ Symptom clusters in acute myocardial infarction: a secondary data analysis Nurs Res 2007 56 2 72 81 10.1097/01.NNR.0000263968.01254.d6 17356437

[B13] Hall SA Cinar A L LC Kopp ZS Roehrborn CG Kaplan SA Rosen RC Do urological symptoms cluster among women? Results from the Boston Area Community Health Survey BJU Int 2008 101 10 1257 1266 10.1111/j.1464-410X.2008.07557.x 18419699

[B14] Thong MS van Dijk S Noordzij M Boeschoten EW Krediet RT Dekker FW Kaptein AA for the NECOSAD Study Group Symptom clusters in incident dialysis patients: associations with clinical variables and quality of life Nephrol Dial Transplant 2008 24 1 225 230 10.1093/ndt/gfn449 18689791

[B15] Hurtig IM Raak RI Kendall SA Gerdle B Wahren LK Quantitative sensory testing in fibromyalgia patients and in healthy subjects: identification of subgroups Clin J Pain 2001 17 4 316 322 10.1097/00002508-200112000-00005 11783811

[B16] Giesecke T Williams DA Harris RE Cupps TR Tian X Tian TX Gracely RH Clauw DJ Subgrouping of fibromyalgia patients on the basis of pressure-pain thresholds and psychological factors Arthritis Rheum 2003 48 10 2916 2922 10.1002/art.11272 14558098

[B17] Hamilton NA Karoly P Zautra AJ Health goal cognition and adjustment in women with fibromyalgia J Behav Med 2005 28 5 455 466 10.1007/s10865-005-9013-8 16179980

[B18] Thieme K Turk D Heterogeneity of psychophysiological stress responses in fibromyalgia syndrome patients Arthritis Res Ther 2006 8 1 R9 10.1186/ar1863 16356200 PMC1526563

[B19] de Souza JB Goffaux P Julien N Potvin S Charest J Marchand S Fibromyalgia subgroups: profiling distinct subgroups using the Fibromyalgia Impact Questionnaire. A preliminary study Rheumatol Int 2008 29 5 509 515 10.1007/s00296-008-0722-5 18820930

[B20] Häuser W Akritidou I Felde E Klauenberg S Maier C Hoffmann A Köllner V Hinz A [Steps towards a symptom-based diagnosis of fibromyalgia syndrome. Symptom profiles of patients from different clinical settings.][German] Z Rheumatol 2008 67 511 515 10.1007/s00393-008-0327-0 18830659

[B21] Arnold LM Russell IJ Diri EW Duan WR Young JP Sharma U Martin SA Barrett JA Haig G A 14-week, randomized, double-blind, placebo-controlled monotherapy trial of pregabalin in patients with fibromyalgia J Pain 2008 9 9 792 805 10.1016/j.jpain.2008.03.013 18524684

[B22] Arnold LM Crofford LJ Mease PJ Burgess SM Palmer SC Abetz L Martin SA Patient perspectives on the impact of fibromyalgia Patient Educ Couns 2008 73 1 114 120 10.1016/j.pec.2008.06.005 18640807 PMC2564867

[B23] Miller GA The magical number seven plus or minus two: some limits on our capacity for processing information Psychol Rev 1956 63 2 81 97 10.1037/h0043158 13310704

[B24] Streiner DL Norman GR Health measurement scales: a practical guide to their development and use 2003 3 New York: Oxford University Press

[B25] Kleinbaum DG Klein M Logistic Regression. A Self-Learning Text 2002 2 New York, NY: Springer-Verlag

[B26] Aldenderfer MS Blashfield RK Cluster Analysis 1984 Newbury Park, CA: Sage Publications, Inc6329508

[B27] Davison ML Multidimensional Scaling 1983 New York, NY: John Wiley & Sons

[B28] SAS Institute SAS/STAT User's Guide. Version 8.2 2001 Cary, NC: SAS Institute

[B29] Scharf MB Baumann M Berkowitz DV The effects of sodium oxybate on clinical symptoms and sleep patterns in patients with fibromyalgia J Rheumatol 2003 30 1070 1074 12734908

[B30] Moldofsky H Alvarez-Horine S Effects of sodium oxybate on sleep physiology and sleep-related symptoms in fibromyalgia. [abstract] Ann Rheum Dis 2008 67 Suppl II 25617604285

[B31] Arnold L Pritchett YL D'Souza DN Goldstein DJ Iyengar S Wernicke JF A randomized, double-blind, placebo-controlled trial of duloxetine in the treatment of women with fibromyalgia with or without major depressive disorder Pain 2005 119 5 15 10.1016/j.pain.2005.06.031 16298061

[B32] Russell IJ Mease PJ Smith TR Kajdasz DK Wohlreich MM Detke MJ Walker DJ Chappell AS Arnold LM Efficacy and safety of duloxetine for treatment of fibromyalgia in patients with or without major depressive disorder: Results from a 6-month, randomized, double-blind, placebo-controlled, fixed-dose trial Pain 2008 136 3 432 444 10.1016/j.pain.2008.02.024 18395345

[B33] Clauw DJ Mease P Palmer RH Gendreau M Wang Y Milnacipran for the treatment of fibromyalgia in adults: a 15-Week, multicenter, randomized, double-blind, placebo-controlled, multiple-dose clinical trial Clin Ther 2008 30 11 1988 2004 10.1016/j.clinthera.2008.11.009 19108787

[B34] Russell IJ Fibromyalgia syndrome: Approach to management Prim Psychiatry 2006 13 9 76 84

[B35] Serra E Duloxetine and pregabalin: safe and effective for the long-term treatment of fibromyaliga? [commentary] Nat Clin Pract Neurol 2008 4 11 594 595 10.1038/ncpneuro0936 18852724

[B36] Mease PJ Seymour K Fibromyalgia: should the treatment paradigm be monotherapy or combination pharmacotherapy? Curr Pain Headache Rep 2008 12 6 399 405 10.1007/s11916-008-0068-4 18973731

[B37] Affleck G Urrows S Tennen H Higgins P Abeles M Sequential daily relations of sleep, pain intensity, and attention to pain among women with fibromyalgia Pain 1996 68 2-3 363 368 10.1016/S0304-3959(96)03226-5 9121825

[B38] McKracken LM Iverson GL Disrupted sleep patterns and daily functioning in patients with chronic pain Pain Res Manag 2002 7 2 75 79 12185371 10.1155/2002/579425

[B39] Smith MT Haythornthwaite JA How do sleep disturbance and chronic pain inter-relate? Insights from the longitudinal and cognitive-behavioral clinical trials literature Sleep Med Rev 2004 8 2 119 132 10.1016/S1087-0792(03)00044-3 15033151

[B40] Moldofsky H Scarisbrick P England R Smythe H Musculosketal symptoms and non-REM sleep disturbance in patients with "fibrositis syndrome" and healthy subjects Psychosom Med 1975 37 4 341 351 169541 10.1097/00006842-197507000-00008

[B41] Bigatti SM Hernandez AM Cronan TA Rand KL Sleep disturbances in fibromyalgia syndrome: Relationship to pain and depression Arthritis Rheum 2008 59 7 961 967 10.1002/art.23828 18576297 PMC3691959

[B42] Burns JW Crofford LJ Chervin RD Sleep stage dynamics in fibromyalgia patients and controls Sleep Med 2008 9 6 689 696 10.1016/j.sleep.2007.10.022 18314389

[B43] Katz RS Heard AR Mills M Leavitt F The Prevalence and Clinical Impact of Reported Cognitive Difficulties (Fibrofog) in Patients With Rheumatic Disease With and Without Fibromyalgia J Clin Rheumatol 2004 10 2 53 58 10.1097/01.rhu.0000120895.20623.9f 17043464

[B44] Rutledge DN Mouttapa M Wood PB Symptom clusters in fibromyalgia: potential utility in patient assessment and treatment evaluation Nurs Res 2009 58 5 359 367 10.1097/NNR.0b013e3181b499d2 19752676

[B45] Harris RE Williams DA McLean SA Sen A Hufford M Gendreau RM Gracely RH Clauw DJ Characterization and consequences of pain variability in individuals with fibromyalgia Arthritis Rheum 2005 52 11 3670 3674 10.1002/art.21407 16258905

[B46] Ahles TA Khan SA Yunus MB Spiegel DA Masi AT Psychiatric status of patients with primary fibromyalgia, patients with rheumatoid arthritis, and subjects without pain: a blind comparison of DSM-III diagnoses Am J Psychiatry 1991 148 12 1721 1726 1957937 10.1176/ajp.148.12.1721

[B47] Mease PJ Arnold LM Crofford LJ Williams DA Russell IJ Humphrey L Abetz L Martin SA Identifying the clinical domains of fibromyalgia: Contributions from clinician and patient delphi exercises Arthritis Rheum 2008 59 7 952 960 10.1002/art.23826 18576290

[B48] Markkula R Järvinen P Leino-Arjas P Koskenvuo M Kalso E Kaprio J Clustering of symptoms associated with fibromyalgia in a Finnish twin cohort Eur J Pain 2008 13 7 744 750 10.1016/j.ejpain.2008.09.007 18938094

[B49] Mease PJ Choy EH Pharmacotherapy of fibromyalgia Rheum Dis Clin North Am 2009 35 2 359 372 10.1016/j.rdc.2009.06.007 19647148

[B50] Wilson HD Robinson JP Turk D Toward the identification of symptom patterns in people with fibromyalgia Arthritis Rheum 2009 61 4 527 534 10.1002/art.24163 19333980 PMC3200227

